# A comparative genomic analysis between methicillin-resistant *Staphylococcus aureus* strains of hospital acquired and community infections in Yunnan province of China

**DOI:** 10.1186/s12879-020-4866-6

**Published:** 2020-02-13

**Authors:** Feng Liao, Zhishuo Mo, Wenpeng Gu, Wen Xu, Xiaoqing Fu, Yunhui Zhang

**Affiliations:** 1 Faculty of Life science and Biotechnology, Kunming University of Science and Technology, Kunming, 650500 People’s Republic of China; 2 The Affiliated Hospital of Kunming University of Science and Technology, Kunming, 650500 People’s Republic of China; 3 Department of Respiratory Medicine, The First People’s Hospital of Yunnan Province, Kunming, 650022 People’s Republic of China; 4 The Third Affiliated Hospital of Sun Yat-Sen University, Guangzhou, 510630 People’s Republic of China; 5 Department of Acute Infectious Diseases Control and Prevention, Yunnan Provincial Centre for Disease Control and Prevention, Kunming, 650022 People’s Republic of China

**Keywords:** Methicillin-resistant *Staphylococcus aureus*, Comparative genome, Genotype, Southwest China

## Abstract

**Background:**

Currently, *Staphylococcus aureus* is one of the most important pathogens worldwide, especially for methicillin-resistant *S. aureus* (MRSA) infection. However, few reports referred to patients’ MRSA infections in Yunnan province, southwest China.

**Methods:**

In this study, we selected representative MRSA strains from patients’ systemic surveillance in Yunnan province of China, performed the genomic sequencing and compared their features, together with some food derived strains.

**Results:**

Among sixty selective isolates, forty strains were isolated from patients, and twenty isolated from food. Among the patients’ strains, sixteen were recognized as community-acquired (CA), compared with 24 for hospital-acquired (HA). ST6-t701, ST59-t437 and ST239-t030 were the three major genotype profiles. ST6-t701 was predominated in food strains, while ST59-t437 and ST239-t030 were the primary clones in patients. The clinical features between CA and HA-MRSA of patients were statistical different. Compared the antibiotic resistant results between patients and food indicated that higher antibiotic resistant rates were found in patients’ strains. Totally, the average genome sizes of 60 isolates were 2.79 ± 0.05 Mbp, with GC content 33% and 84.50 ± 0.20% of coding rate. The core genomes of these isolates were 1593 genes. Phylogenetic analysis based on pan-genome and SNP of strains showed that five clustering groups were generated. Clustering ST239-t030 contained all the HA-MRSA cases in this study; clustering ST6-t701 referred to food and CA-MRSA infections in community; clustering ST59-t437 showed the heterogeneity for provoking different clinical diseases in both community and hospital. Phylogenetic tree, incorporating 24 isolates from different regions, indicated ST239-t030 strains in this study were more closely related to T0131 isolate from Tianjin, China, belonged to ‘Turkish clade’ from Eastern Europe; two groups of ST59-t437 clones of MRSA in Yunnan province were generated, belonged to the ‘Asian-Pacific’ clone (AP) and ‘Taiwan’ clone (TW) respectively.

**Conclusions:**

ST239-t030, ST59-t437 and ST6-t701 were the three major MRSA clones in Yunnan province of China. ST239-t030 clonal Yunnan isolates demonstrated the local endemic of clone establishment for a number of years, whereas ST59-t437 strains revealed the multi-origins of this clone. In general, genomic study on epidemic clones of MRSA in southwest China provided the features and evolution of this pathogen.

## Background

*Staphylococcus aureus* is the most commonly isolated pathogen from human and often lead to endovascular infections, endocarditis, osteomyelitis, septic arthritis and pneumonia [1]. Specifically, methicillin-resistant *S. aureus* (MRSA) infection has exploded in both healthcare facility and community since 1990s [2]. Although the availability of antimicrobial agents to treat MRSA infections, it still to be the dominant cause of mortality and morbidity all over the world [3]. The spread of different clones from different geographic regions have been reported, MRSA has been verified as one of the most important pathogen in hospital or community setting [4, 5]. Hospital-acquired MRSA (HA-MRSA) infections often lead to ventilator associated pneumonias, intravenous catheter associated infections or surgical wound infections, while community-acquired MRSA (CA-MRSA) infections commonly cause skin and soft tissue infection, sometimes are invasive and life threatening [6, 7].

The whole genome sequencing method has become the gold standard for understanding the genetic diversity of bacteria [8]. Nowadays, large numbers of genomic reports have revealed the molecular epidemiological consequences of clone changes or different infectious syndromes in *S. aureus*, especially for MRSA [9, 10]. Thus, study on genomic level of MRSA will promote the understanding of the evolution and biology of this pathogen, improving the prevention and management of MRSA. The phylogenetic investigation revealed the dissemination and hospital transmission of ST239 clone through Europe, North America, South America and Asia [11]. Therefore, ST239 clone of strains was the most important HA-MRSA around the world. However, ST59 clone of isolates were the primary CA-MRSA infections in Asia [12, 13]. Furthermore, our previous study [14] on molecular characteristics of *S. aureus* from food surveillance indicated that ST6-t701 was the prevalent genotypes in southwest China, and some strains had the same genotype profiles with patients’ isolates. The different clonal structures of MRSA strains maybe represent the different infection sources or disease spectrums. In this study, we selected representative MRSA strains from patients’ systemic surveillance in Yunnan province of China, performed the genomic sequencing and compared their features, together with some food derived strains.

## Methods

### Bacterial source and case definitions

A total of 60 MRSA strains were selected from 2013 to 2016 in both patients and food isolates in Yunnan province, southwest China. All strains were collected consecutively during the same period in both hospital and food surveillance. CA-MRSA strains of patients were isolated from The First People’s Hospital of Yunnan province (hospital A, HA). In contrast, HA-MRSA were isolated from People’s Hospital of Kunming City (hospital B, HB). CA-MRSA case was defined as MRSA isolated within 48 h after admission without the following risk factors: history of hospitalization, surgery, dialysis or stay in a long-term care unit within 1 year; dependence on an indwelling catheter, intravenous line or a percutaneous device when cultured; or previous isolation of MRSA. HA-MRSA cases were defined as MRSA isolation within 48 h with at least one risk factor, as mentioned above, or beyond 48 h, regardless of risk factors [15]. Hospitals A and B are tertiary care academic medical centers in southwest China and cover the entire Kunming area. The basic clinical information of patients from two hospitals were collected. For all these patients’ isolates, we have performed pulsed field gel electrophoresis (PFGE), multilocus sequence typing (MLST) and *spa* typing and selected them from previous systemic surveillance results (Additional files 1 and 2). The selection criterions of strains in this study were shown as follows: a. three major clones of patients’ MRSA, including ST239-t030, ST59-t437 and ST6-t701 were involved in the study, also contained some highly similarity strains with food derived strains (ST188-t189 and ST965-t062 etc); b. ST239-t030, ST59-t437, ST6-t701 and ST188-t189 generated four cluster groups based on PFGE patterns (Additional file 1), and we selected different PFGE pattern strains for sequencing in each genotype profile, except some highly similar isolates with food strains, such as seven ST59-t437, four ST6-t701 and three ST965-t062 strains.

### Antibiotic-resistant test

Minimum inhibitory concentrations (MICs) for the 13 antibiotics were determined through the broth microdilution method using customized microtiter plates (Sensititre, UK) according to the manufacturers’ instructions. The antibiotics tested were penicillin (PCN), oxacillin (OXA), gentamicin (GEN), ciprofloxacin (CIP), levofloxacin (LEV), moxifloxacin (MXF), erythromycin (EM), clindamycin (CM), linezolid (LZD), vancomycin (VAN), tetracycline (TET), rifampicin (RIF) and trimethoprim/sulfamethoxazole (SXT). The tests were performed and interpreted in accordance with the Clinical and Laboratory Standards Institute (CLSI) guidelines (M100, 2018) [16], and *S. aureus* ATCC 29213 was used as a quality control. The *mec* gene was detected by PCR as described by previous study [17].

### DNA extraction and genome sequencing

All MRSA isolates were recovered on Brain Heart Agar (BHI) (Luqiao, Beijing) at 37 °C for 24 h. Total genomic DNA of the isolated bacteria was extracted using a bacterial total genomic DNA extraction kit (Tiangen, Beijing) following the manufacturer’s instructions. All DNA samples were stored at − 20 °C for complete genome analysis.

Bacterial genome sequencing of all isolates was performed by our laboratory on the Illumina HiSeq platform using 2 × 150 bp paired-end reads. The libraries were built using a Nextera XT DNA Library Prep Kit following the manufacturer’s reference guidelines. Generally, 1 ng genomic DNA of each strain was used. After segment and purification, index PCR was performed to add the Illumina Nextera barcodes using i5 and i7 primers, and then the purification was executed again to remove non-target fragments. Finally, the libraries were normalized, pooled and sequenced using an Illumina HiSeq sequencing system (Illumina, San Diego, USA).

### Assembly and annotation

The raw data were trimmed for quality control, and low-quality (<Q40) reads were filtered by Trimmomatic (version 0.38) [18]. Draft genomes were assembled using SOAPdenovo (version 2.04), with k-mer values (25, 31, 37, 47, 59, 71, 83 and 95) optimized to the best assembly results [19, 20]. Gapcloser (version 1.12r6) was used to fill the genomic gaps. GenemarkS software (version 4.28) [21] with default parameters was used to predict the open reading frame (ORF) of each genome, and the predicted amino acid sequences were aligned and annotated by DIAMOND [22] (E-value: 1e-5, top 5) to NCBI non-redundant nucleotide database (NR), SwissProt, TrEMBL, KEGG, GO, COG, CDD, Pfam, Pathogen-Host Interaction database (PHI), Antibiotic Resistance Genes Database (ARDB), and Virulence Factor Database (VFDB).

### Comparative genomic analyses

We performed core- and pan-genome analysis using PGAP [23], which employed an all-versus-all BLAST search to group all CDSs into homologous clusters with default settings. All of the 60 strains sharing CDSs were considered to be core genes, and CDSs without orthologs were considered to be unique genes. Orthologs Clusters were annotated in COG functional distributions. Twenty-four MRSA references from other global strains in publically available datasets were used for phylogenetic analysis to determine the dissemination and evolution of these clonal lineages in southwest China (Additional file 3). Pairwise colinear comparisons of all genome sequences were performed using Mummer3 [24]. The pan-based tree was generated based on the presence or absence of genes in the pan-genome using the neighbor-joining method, and the SNP-based tree was generated based on the total genomic sequence results by using neighbor-joining method as well. The phylogenetic trees were visualized and ordered with FigTree (version 1.4.3) and iTol.

### Statistics

Statistical analysis was performed using the SPSS software package (version 16.0, IBM, USA). χ2 or Kruskal-Wallis H test were used if appropriate. A *P* value of < 0.05 was recognized as statistically significant.

The sequence data have been deposited into the National Center for Biotechnology Information (NCBI), https://www.ncbi.nlm.nih.gov/ with accession numbers VCEL00000000 to VCGT00000000 (PRJNA543691).

## Results

### General information of MRSA in this study

**Fig. 1:**
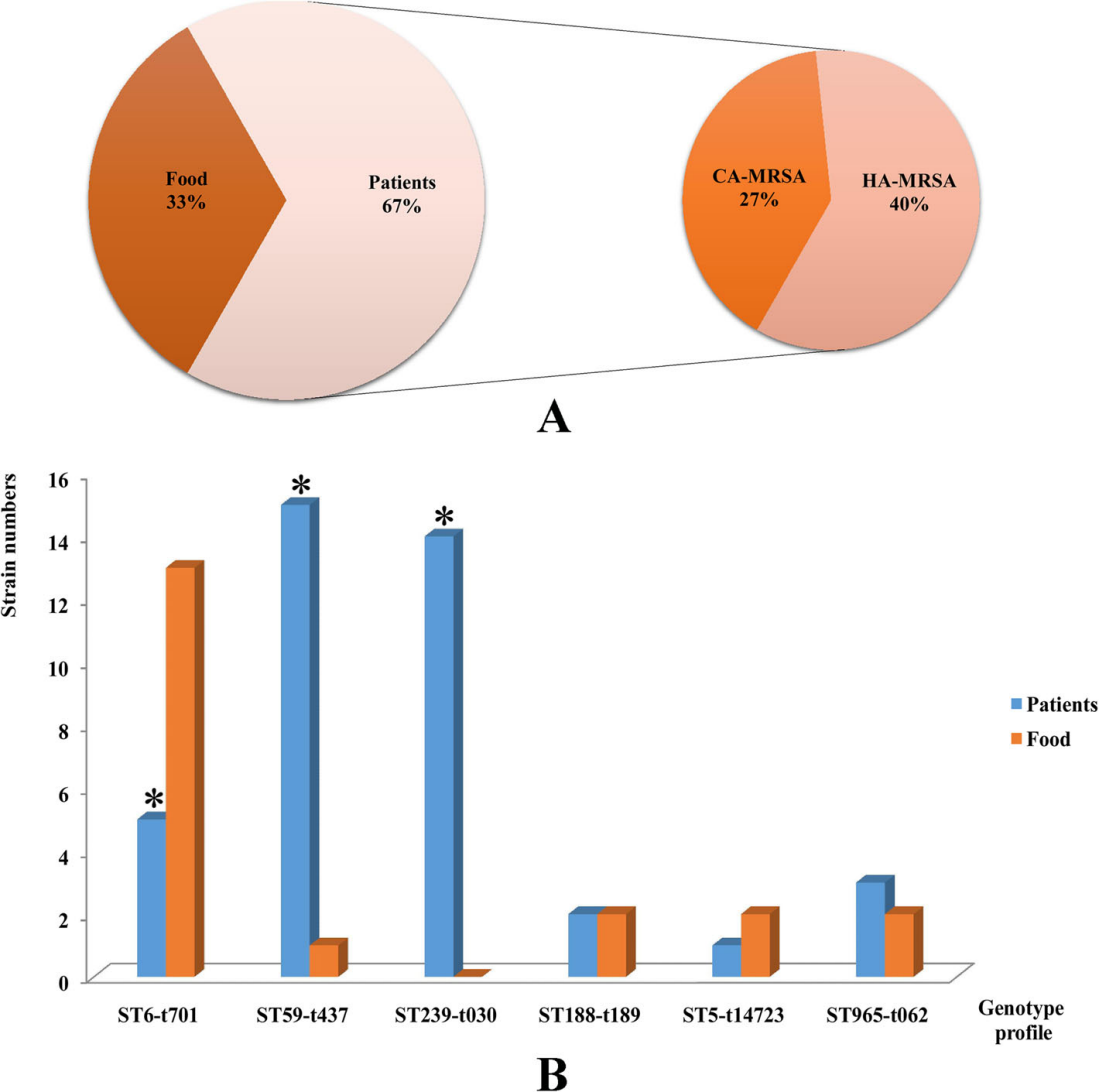
The source and genotype profiles of MRSA used in this study. **a** The source of MRSA strains. **b** The distributions of genotype profiles of the isolates (* indicated the distribution of genotypes between patients and food was statistically significant)

For all the MRSA used in this study, forty strains (66.7%) were isolated from patients, and 20 (33.3%) isolated from food. Among the patients’ strains, sixteen (27%) were recognized as CA-MRSA, compared with 24 (40%) for HA-MRSA (Fig. 1a). ST6-t701 (18, 30%), ST59-t437 (16, 26.7%) and ST239-t030 (14, 23.3%) were the three major genotype profiles in this study. Other genotypes contained ST965-t062, ST188-t189 and ST5-t14723, as Fig. 1b shown. ST6-t701 was predominated in food strains, while ST59-t437 and ST239-t030 were the primary clones in patients (Fig. 1b). The comparison of clinical features between CA and HA-MRSA from patients revealed that except for sex, the factors age, clinical manifestations, and sample types were statistically different (*P* < 0.05), as Table 1 shown. Most of the CA-MRSA cases were children from pediatrics under the 5 years. The genotype profiles of CA-MRSA strains showed highly heterogeneity (Simpson’s index = 0.750) compared with HA-MRSA (Simpson’s index = 0.487). All the genotypes could be found in CA-MRSA isolates, except ST239-t030. However, the HA-MRSA cases were mostly from old patients, and the patients had different primary diseases, such as hypertension, cardiovascular disease, trauma, tumor or other infections. 58.3% of HA-MRSA were ST239-t030, and 41.7% were ST59-t437 genotype. All the food strains were isolated from eight cities of Yunnan province, and the majority (21.7%) was isolated from grain products. The details of MRSA strains information in this study were shown in additional file 4.

**Table 1:** Clinical features between community- and hospital-acquired MRSA in this study

Parameters		Groups				χ2	*P* value
		CA-MRSA	%	HA-MRSA	%		
Sex	Male	9	56.2	14	58.3	0.017	0.896
	Female	7	43.8	10	41.7		
Age groups	≤5 years	12	75	0	0	33.52	0.000
	5–18 years	2	12.5	0	0		
	18–50 years	2	12.5	7	29.2		
	>50 years	0	0	17	70.8		
Clinical manifestation	Fever and pneumonia	6	37.5	10	41.7	24.38	0.000
	Respiratory symptoms (cough or expectoration)	10	62.5	0	0		
	Trauma	0	0	3	12.5		
	Tumor	0	0	3	12.5		
	Infections or others	0	0	8	33.3		
Sample types	Sputum	5	31.2	15	62.5	22.29	0.000
	Nasopharyngeal aspiration	10	62.5	0	0		
	Blood	1	6.2	1	4.2		
	Secreta	0	0	8	33.3		
Genotype profiles	ST6-t701	5	31.2	0	0	26.11	0.000
	ST59-t437	5	31.2	10	41.7		
	ST239-t030	0	0	14	58.3		
	ST188-t189	2	12.6	0	0		
	ST5-t14723	1	6.2	0	0		
	ST965-t062	3	18.8	0	0		

### Antibiotic resistant results

All the MRSA in this study were resistant to OXA and PCN, but sensitive to VAN. The *mec* gene was positive for all the isolates. In general, 55% of all the strains were resistant to MXF, followed by SXT (41.7%) and EM (36.7%). Compared the antibiotic resistant results between patients and food indicated that higher antibiotic resistant rates were found in patients’ strains, such as LZD, CIP, LEV, RIF, TET and MXF (Table 2). The antibiotic resistant results between genotypes of strains were also different. ST59-t437 genotype strains showed higher antibiotic resistant rates in CM, CIP, EM, SXT and TET, while ST239-t030 had higher resistant rate in MXF (*P* < 0.05). ST6-t701 clone had the lowest resistant rate for most of antibiotics, as Table 2 shown.

**Table 2:** The antibiotic resistant results in this study

Antibiotics	Interpretation	Groups				χ2	*P* value	Groups								χ2	*P* value
		Patient	%	Food	%			ST6-t701	%	ST59-t437	%	ST239-t030	%	Others	%		
LZD	R	11	27.5	0	0	6.74	0.009	4	22.2	4	25	0	0	3	25	4.16	0.245
	S	29	72.5	20	100			14	77.8	12	75	14	100	9	75		
CM	R	7	17.5	4	20	4.29	0.117	2	11.1	6	37.5	1	7.1	2	16.7	14.57	0.024
	S	2	55	15	75			16	88.9	5	31.2	9	64.3	7	58.3		
	I	11	27.5	1	5			0	0	5	31.2	4	28.6	3	25		
CIP	R	13	32.5	5	25	5.18	0.075	4	22.2	6	37.5	2	14.3	6	50	15.99	0.014
	S	7	17.5	0	0			0	0	5	31.2	2	14.3	0	0		
	I	20	50	15	75			14	77.8	5	31.2	10	71.4	6	50		
EM	R	14	35	8	40	7.09	0.029	5	27.8	11	68.8	1	7.1	5	41.7	30.63	0.00
	S	15	37.5	12	60			13	72.2	1	6.2	6	42.9	7	58.3		
	I	11	27.5	0	0			0	0	4	25	7	50	0	0		
GEN	R	4	10	0	0	4.64	0.098	0	0	2	12.5	2	14.3	0	0	28.9	0.00
	S	26	65	18	90			17	94.4	13	81.2	3	21.4	11	91.7		
	I	10	25	2	10			1	5.6	1	6.2	9	64.3	1	8.3		
LEV	R	19	47.5	0	0	13.95	0.001	5	27.8	5	31.2	3	21.4	6	50	6.6	0.36
	S	8	20	7	35			4	22.2	6	37.5	2	14.3	3	25		
	I	13	32.5	13	65			9	50	5	31.2	9	64.3	3	25		
RIF	R	2	5	0	0	9.13	0.01	0	0	1	6.2	1	7.1	0	0	43.03	0.00
	S	26	65	20	100			18	100	14	87.5	2	14.3	12	100		
	I	12	30	0	0			0	0	1	6.2	11	78.6	0	0		
SXT	R	18	45	7	35	0.55	0.459	5	27.8	7	43.8	4	28.6	9	75	7.93	0.047
	S	22	55	13	65			13	72.2	9	56.2	10	71.4	3	25		
TET	R	13	32.5	2	10	9.57	0.008	2	11.1	10	62.5	2	14.3	1	8.3	28.47	0.00
	S	20	50	18	90			16	88.9	3	18.8	8	57.1	11	91.7		
	I	7	17.5	0	0			0	0	3	18.8	4	28.6	0	0		
MXF	R	32	80	1	5	30.3	0.00	5	27.8	9	56.2	12	85.7	7	58.3	10.79	0.013
	S	8	20	19	95			13	72.2	7	43.8	2	14.3	5	41.7		

The molecular determinants based on ARDB annotations revealed several resistant genes were commonly found between these strains, such as *liaR*, *mprF*, *pgsA* and *rpoC* resistant to daptomycin; *UhpT*, *murA* and *GlpT* resistant to fosfomycin; *gyrA* and *parC* resistant to fluoroquinolones; *gyrB* and *parE* resistant to aminocoumarin; *rpoB* resistant to rifampicin; *rpsL* resistant to Streptomycin. The majorities of *ErmC*, *ErmT*, *ErmY*, *ErmG*, *ErmA*, *ErmB* resistant to erythromycin and *tetM*, *tetO*, *tetS*, *tet32*, *tetW*, *tetQ* resistant to tetracycline were found in ST59-t437 isolates.

### Genome features

Totally, 682,961,826 raw reads were obtained for all the MRSA strains, 643,281,744 valid reads were retained after quality control and the effective rate was 94.19%. The average genome sizes of MRSA were 2.79 ± 0.05 Mbp, with GC content 33% (Additional file 5). The average predicted genes were 2693.35 ± 65.19, with average length 875.05 ± 7.04 (bp), and the coding rate of all predicted genes were 84.50 ± 0.20% for all MRSA in this study. In addition, 50.72 ± 8.38 average tRNA-coding genes and 7.27 ± 0.92 average rRNA operons were identified. The average sequencing depth was 548.48 ± 92.51, and the average coverage of genome was 93.79 ± 1.28%, as Additional file 5 shown. In general, 2572.03 ± 60.90, 2122.35 ± 34.99, 2037.05 ± 16.12, and 2571.48 ± 60.81 average genes were annotated in NR, PFAM, Swissprot and TrEMBL database respectively. 1896.53 ± 17.79 and 1484.57 ± 8.98 average genes were annotated in GO and KEGG pathways, while 1963.68 ± 24.11 average genes were annotated in COG categories.

### Core and pan-genome analysis

**Fig. 2:**
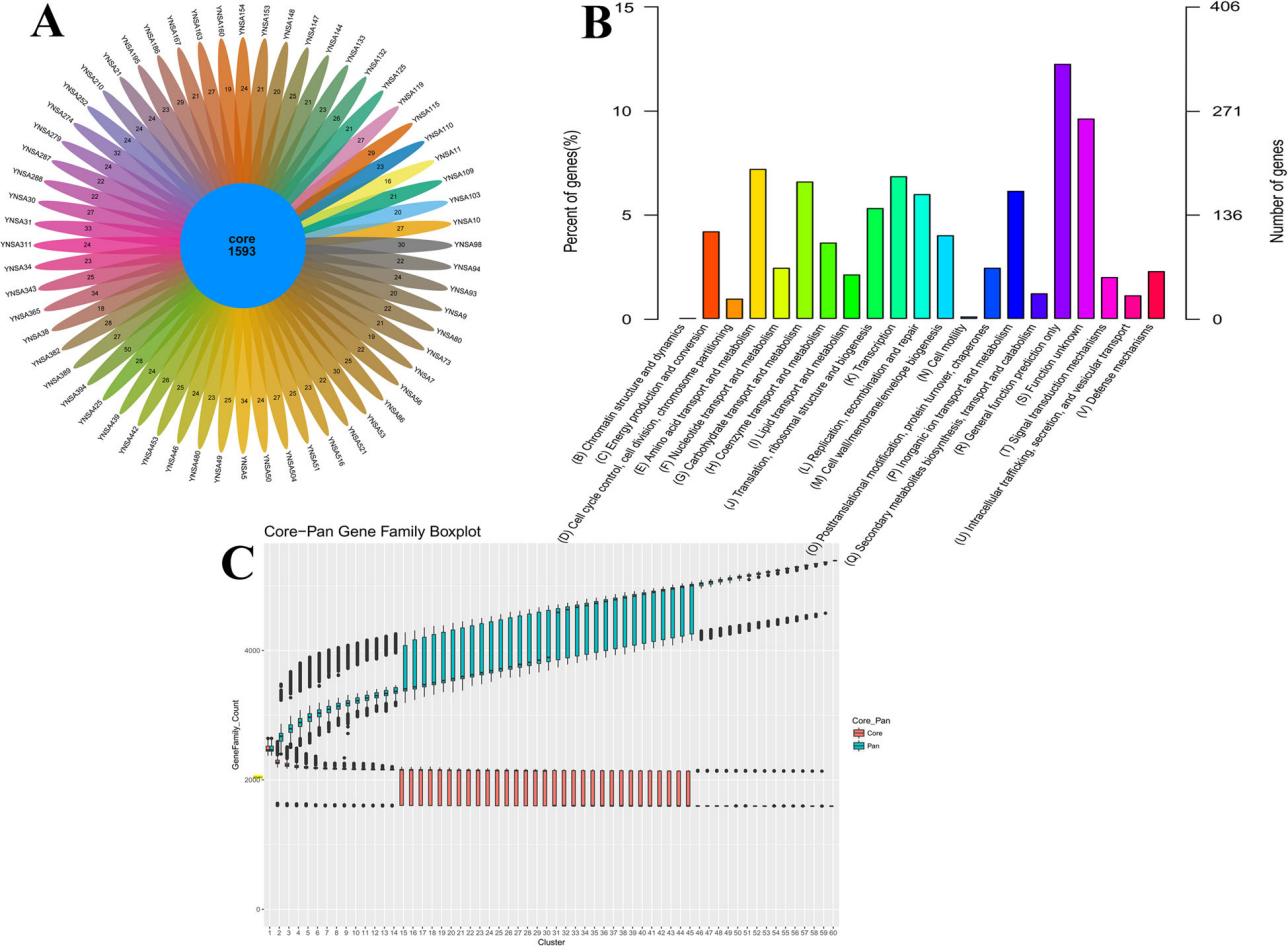
The core and pan-genome analyses of 60 MRSA in this study. **a**. The flower diagram of numbers of orthologs cluster for all the strains. The core number indicated the genes shared with all bacteria. Other numbers represented the specific genes in each MRSA strain. **b** COG categories distributions of whole orthologs cluster. **c** Core-pan gene family boxplot in this study

All CDSs from all MRSA sequenced in this study were used for core and pan-genome analyses. Core genes were considered as the most conserved and shared by all strains, whereas dispensable genes were shared by unique genes identified in only one of the genomes. The most of the CDSs were highly conserved among MRSA strains, and the core genomes of these isolates were 1593 genes (Fig. 2a). Dispensable genes were distributed ranging from 16 to 50 genes in different strains (Fig. 2a). According to homologous whole clusters COG expansions, 1040 genes were assigned to metabolism, e.g., E (amino acid transport and metabolism), G (carbohydrate transport and metabolism) and P (inorganic ion transport and metabolism). Five hundred sixty-four genes were assigned to information storage and processing category, e.g., K (transcription), L (replication, recombination and repair) and J (translation, ribosomal structure and biogenesis). Three hundred ninety-six genes were assigned to cellular processes and signaling, e.g., M (cell wall/membrane/envelope biogenesis), O (posttranslational modification, protein turnover, chaperones) and V (defense mechanisms), as Fig. 2b shown. Generally, the size of the core and pan-genome depended on the number of strains were considered in the analyses. It was evident that the pan-genome curve converges rapidly, indicating the variation in MRSA strains. However, the core genome size was stable, decreasing initially and reached a plateau at 2000 genes (Fig. 2c). This revealed that MRSA has an open pan-genome in this study.

### Phylogenetic analysis

**Fig. 3:**
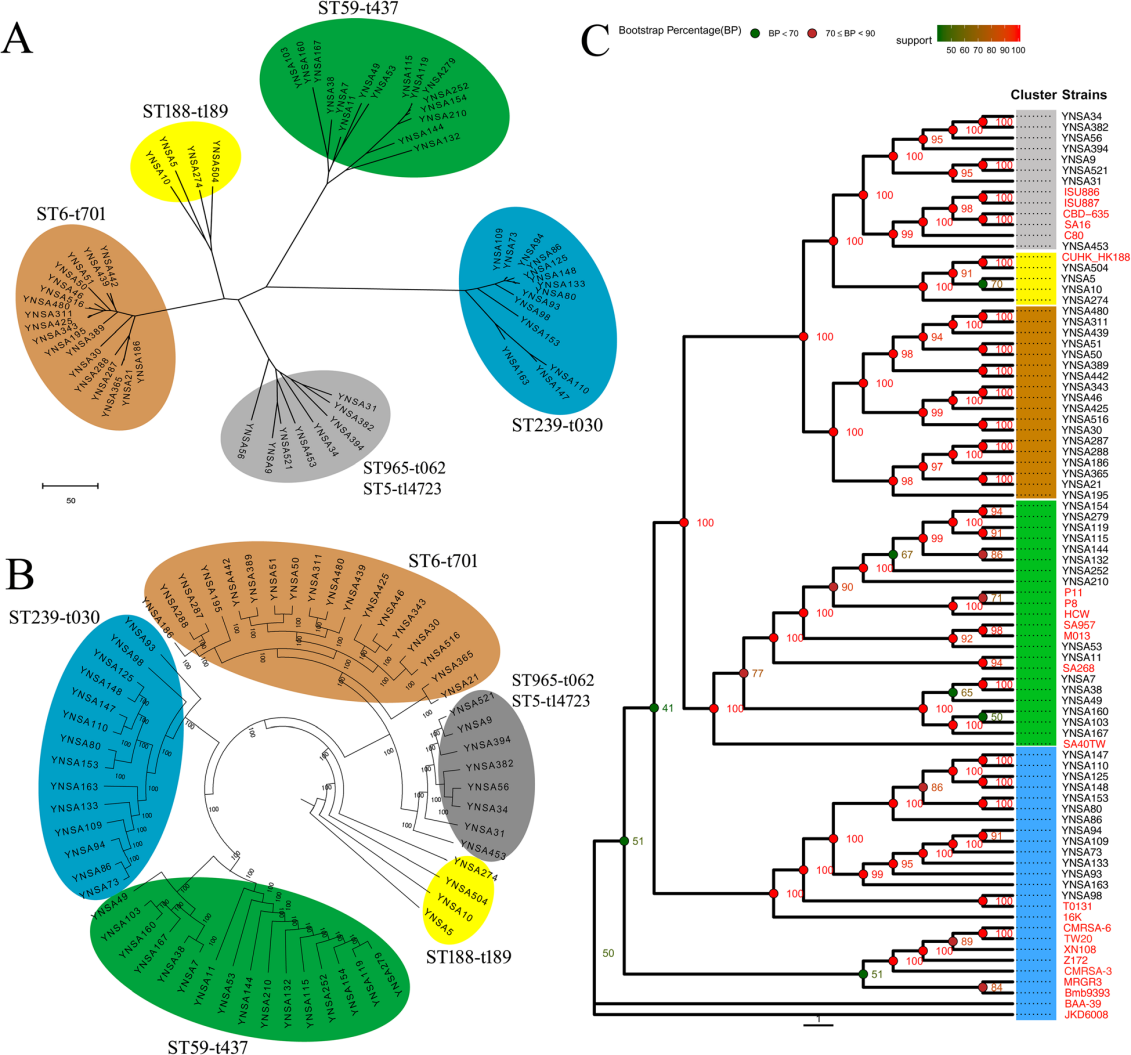
Phylogenetic trees of MRSA used in this study. **a** Pan-genome based neighbor-joining tree of all the MRSA. ST6-t701, clustering brown; ST59-t437, clustering green; ST239-t030, clustering blue; ST188-t189, clustering yellow; ST965-t062 and ST5-t14723, clustering gray. **b** SNP based neighbor-joining tree of all the MRSA. ST6-t701, clustering brown; ST59-t437, clustering green; ST239-t030, clustering blue; ST188-t189, clustering yellow; ST965-t062 and ST5-t14723, clustering gray. **c** Pan-genome based neighbor-joining tree of 84 MRSA, containing 60 strains in this study and 24 references. ST6-t701, clustering brown; ST59-t437, clustering green; ST239-t030, clustering blue; ST188-t189, clustering yellow; ST965-t062 and ST5-t14723, clustering gray. The reference strains were shown with red font

Phylogenetic analysis based on pan-genome (core and dispensable genes) of MRSA showed that five clustering groups were generated with brown, yellow, green, blue and gray color (Fig. 3a). Interestingly, the different genotype or clones of strains clustered into different groups, thus, clustering group brown was ST6-t701 strains, green was ST59-t437 isolates, blue was ST239-t030, yellow was ST188-t189, gray was ST965-t062 and ST5-t14723 clone. The SNP based phylogenetic tree also generated identical five clustering groups showing with different color in this study (Fig. 3b). Pan-based phylogenetic tree of 84 MRSA strains (60 isolates in this study and 24 references) revealed that different clusters were generated according to the genotype profiles as mentioned above (Fig. 3c). All the ST239-t030 strains in this study were more closely related to T0131 isolate from Tianjin, China, belonged to ‘Turkish clade’ from Eastern Europe, but far away from TW20 of ‘Asian clade’ or other global reference isolates. Two groups of ST59-t437 clones of MRSA in Yunnan province were generated with some reference strains. YNSA7 to YNSA167 group was more closely related to SA40TW isolate, belonged to the ‘Asian-Pacific’ clone (AP), whereas YNSA154 to YNSA210, YNSA53 and YNSA11 group were closely related to SA957, M013 and SA268 strains, belonged to the ‘Taiwan’ clone (TW). Most of the CC5 clonal MRSA (ST5 and ST965 clone; YNSA34 to YNSA31) were separated from reference strains except YNSA453, as Fig. 3c shown.

**Fig. 4:**
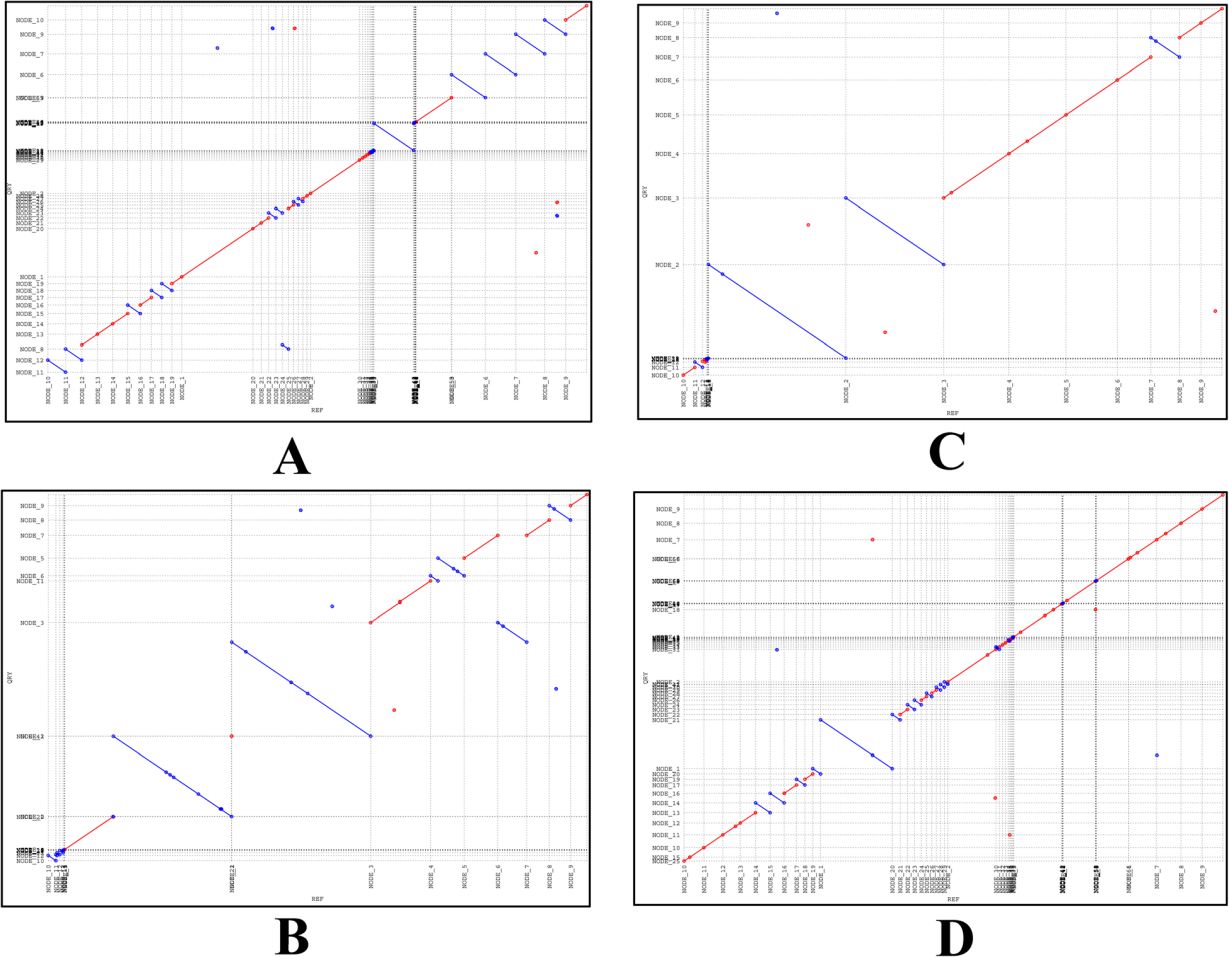
Colinear comparison of MRSA strains in this study. **a** Colinear comparison of YNSA73 and YNSA94. The red line and blue line showed matches that were indicated by the upward and downward slopes, respectively. **b** Colinear comparison of YNSA21 and YNSA365. The red line and blue line showed matches that were indicated by the upward and downward slopes, respectively. **c** Colinear comparison of YNSA311 and YNSA480. The red line and blue line showed matches that were indicated by the upward and downward slopes, respectively. **d** Colinear comparison of YNSA115 and YNSA279. The red line and blue line showed matches that were indicated by the upward and downward slopes, respectively

Clustering ST239-t030 clone comprised all the HA-MRSA cases in this study; it was the primary clone for hospital acquired infection in southwest China. Colinear comparison of strains YNSA73 and YNSA94 in this genotype showed that the chromosome of YNSA73 was collinear with YNSA94 with 99.73% identity (Fig. 4a). ST6-t701 clone (clustering brown) mainly referred to food related diseases in community, since the patients and food strains clustered into identical group, and close similarities could be found among these strains. In this group, YNSA21 was a patient isolates, and YNSA365 was a food strain, the chromosome of YNSA21 was collinear with YNSA365 showing 99.71% identity (Fig. 4b). In addition, YNSA311 and YNSA480 were both isolated from food strains of different sources, their genomes were also collinear with each other showing 99.76% identity (Fig. 4c). ST59-t437 clone represented the most heterogeneous cluster in this study. The CA and HA-MRSA cases both contained ST59-t437 genotype, and some isolates from patients were related with food. Colinear comparison between YNSA115 (food) and YNSA279 (patient) revealed the 99.79% identity of two isolates (Fig. 4d). Therefore, ST59-t437 clone had a high degree of diversity for provoking different clinical spectrum of diseases in both community and hospital.

## Discussion

Studies on MRSA epidemic or outbreaks have originated from developed countries in Europe, North America and Japan [3, 25, 26]. However, MRSA infections became serious problem in developing countries at present, such as China and India. In China, 60% of *S. aureus* infections were caused by MRSA [27], among them, ST239 was the most abundant clone for hospital infections. In addition, several MRSA researches reported ST239 isolates recovered from hospital patients in Asia, South America and Eastern Europe [28–32]. Therefore, ST239 strain may represent the predominant MRSA clone lineage for hospital infections worldwide. In this study, we found ST239-t030 was the major clone for hospital infection in southwest China. This strain was prevalent in the local health-care facilities, and it had also been isolated from different departments within the hospitals. Consequently, the strain ST239-t030 should be treated as the most important MRSA clone by the pathogen surveillance and hospital infection and prevention control teams.

In Asian countries, specifically in China, ST239 clone has been identified as the predominant HA-MRSA at present [33]. Previous study showed that ST239 MRSA lineage at least contained five clades worldwide, such as Europe and Australia, North and South America, and Asian clade [34]. In this study, several published ST239 clonal MRSA complete genomes were used for comparison, namely T0131, TW20 and JKD6008. Specifically, T0131 reference strain was isolated from old patient in Tianjin, China in 2006, and closely related to the strains of the ‘Turkish clade’ and the ‘Russia variant’ (16 K) [35]. Wang et al. [36] compared the genomics of MRSA from Beijing and Hong Kong; they revealed that strains from HK clustered into the ‘Asian clade’, while BJ isolates were clustered with strains of the ‘Turkish clade’. They concluded that origins of ST239 lineage in southern and northern China were different. In our study, Yunnan isolates from southwest China were closely related to T0131 and clustered with strains of the ‘Turkish clade’ from Eastern Europe. We considered the similar ST239-t030 clonal Yunnan isolates in this study were more likely to demonstrate the local endemic of primary clone establishment for a number of years. The result also indicated the possibility that ST239-t030 MRSA from Yunnan originated from a common recent ancestor and spread during this period.

Our previous study [14] compared the *S. aureus* isolates from patients and food, the results showed some strains from patients had identical PFGE patterns, ST and *spa* types with food isolates. These strains were dominated with ST6-t701, ST5-t14723, ST59-t437 and ST965-t062, specifically for ST6-t701. Currently, ST6-t701 strain has become the predominant clone for Staphylococcal food poisoning (SFP) in China. Yan et al. [37] investigated seven outbreaks of 52 *S. aureus* in Shen-zhen, China. They found 63.5% of all the strains belonged to ST6-t701 genotype. Li et al. [38] performed the molecular epidemiology of seven SFP outbreaks caused by *S. aureus* in northwest China; the results also indicated ST6-t701was the primary clone, followed by ST5-t002 and ST59-t172. All these evidences suggested that ST6-t701 of *S. aureus* has an important role in SFP in China. Similar results could be found in our study, ST6-t701 clone was the most important MRSA for food related diseases in Yunnan province. The close genomic relations between food isolates with patients’, and the similarity among different sources of strains in this study both indicated that ST6-t701 was related to foodborne disease in southwest China.

CA-MRSA were characterized by a diversity of clones at the early stage, and then numbers of predominant clones have established around the world. In the United States, the USA300 epidemic clone emerged as major CA-MRSA strain over the past decades [39]. Molecular typing further identified this isolate as ST8, and most commonly *spa* type t008. Other studies reported that highly virulent ST80 strain dominated in Europe [40], and ST93 MRSA clone in Australia [41]. All these studies indicated the diversity of CA-MRSA strains from different regions of the world. Recently, the genome sequence of predominant CA-MRSA ST59 isolate was reported in Taiwan, these strains were frequently multi-drug resistant isolates [42]. Li et al. [43] performed the molecular typing of clonal complex 59 MRSA in seven major cities across mainland China. Their results revealed ST59-t437 was the predominant genotype, 81.8% of the strains were CA-MRSA, and 18.2% were HA-MRSA. They concluded CC59 MRSA could lead to CA and HA infections and the majority of infections were in children. The genomic analysis of CA-MRSA strains in our study also showed highly genetic diversity of isolates. ST6-t701, ST59-t437, ST188-t189, ST5-t14723 and ST965-t062 were all referred to CA-MRSA cases. Specifically, ST59-t437 clone exhibited the multiple roles in MRSA infections, such as community acquired, hospital acquired and even related with food strain.

ST59 clonal MRSA has been prevalent in the Asia-Pacific region, including mainland China, Taiwan, Vietnam, Japan and Singapore [13]. Previous studies revealed two ST59 clones of MRSA were circulated in Taiwan: the ‘Taiwan’ clone (TW) and the ‘Asian-Pacific’ clone (AP) [44, 45]. TW clone caused sepsis and infection, especially in children, whereas AP clone usually colonized as commensal in healthy individuals. Particularly, reference SA957 and SA40 isolates were the typical strains for TW and AP clone respectively, according to several studies [44, 46]. On the mainland China, ST59 was also the major MRSA clone; ST59-t437 was the predominant clone among CA-MRSA on the Chinese mainland [43]. Several studies have been reported the complete genomic analysis of representative strains isolated from patients. For example, the reference SA268 [47], a ST59 CA-MRSA strain, was isolated from a case of pneumonia in a young patient in Zhejiang Province of China; the genome analysis revealed it was almost identical to that of the Taiwanese ST59 CA-MRSA strains SA957 and M013. Cheng et al. [48] investigated an outbreak of CA-MRSA in Hong Kong by using whole genome sequencing. P8, P11 and HCW strains were analyzed in their study, and the results revealed complicated transmission between patients, healthcare worker and environment. In our study, two groups of ST59-t437 clones of MRSA in Yunnan province were generated. One group was more closely related to AP clone, whereas another was closely related to TW clone, and the result probably supported the multi-origin theory of CA-MRSA.

This study was the systemic surveillance for MRSA by using whole genome sequencing in Yunnan province, southwest China, and it gave us an eye on MRSA infections both in hospital and community in local areas. However, several limitations were found in this study. Firstly, strains used in this study were selected from our previous molecular typing database, not all the surveillance isolates were sequenced and analyzed; perhaps there was selection bias of the strains. Secondly, the short surveillance period of time for MRSA in Yunnan province only reflected the prevalence during these years. Thirdly, the scale of MRSA monitoring was relatively limited; only two hospitals were involved in this study. Therefore, further studies referred to strains from older collections and across other parts of southwest China, transmission between communities and hospitals, and even the longitudinal surveillance were required to evaluate the widespread or origin of the MRSA in Yunnan province, southwest China.

## Conclusions

In this study, a comparative genomic analysis was performed between CA and HA-MRSA infections in southwest China. ST239-t030, ST59-t437 and ST6-t701 were the three major MRSA clones in Yunnan province of China. ST239-t030 clonal Yunnan isolates demonstrated the local endemic of clone establishment for a number of years, whereas ST59-t437 strains revealed the multi-origins of this clone. In general, genomic study on epidemic clones of MRSA in southwest China provided the features and evolution of this pathogen.

## Supplementary information

**Additional file 1.** The molecular typing database of patients’ MRSA in Yunnnan province of China.

**Additional file 2.** The genotype profiles of patients’ MRSA in Yunnan province of China.

**Additional file 3.** The details of reference strains used for phylogenetic analysis in this study.

**Additional file 4.** The details of strain information and antibiotic resistant results in this study.

**Additional file 5.** The genome features of 60 MRSA in this study.

## Data Availability

All data generated or analysed during this study are included in this published article. The sequence data have been deposited into the National Center for Biotechnology Information (NCBI), https://www.ncbi.nlm.nih.gov/ with accession numbers VCEL00000000 to VCGT00000000 (PRJNA543691).

## Abbreviations

CA: community-acquired
HA: hospital-acquired
MLST: multilocus sequence typing
MRSA: methicillin-resistant *Staphylococcus aureus*
PFGE: Pulsed-field gel electrophoresis
