# Supplementary figures and images for: A comparative genomic analysis between methicillin-resistant Staphylococcus aureus strains of hospital acquired and community infections in Yunnan province of China
Source: BMC Infect Dis. 2020 Feb 13;20:137. doi: 10.1186/s12879-020-4866-6 (PMC7020539; doi:10.1186/s12879-020-4866-6)

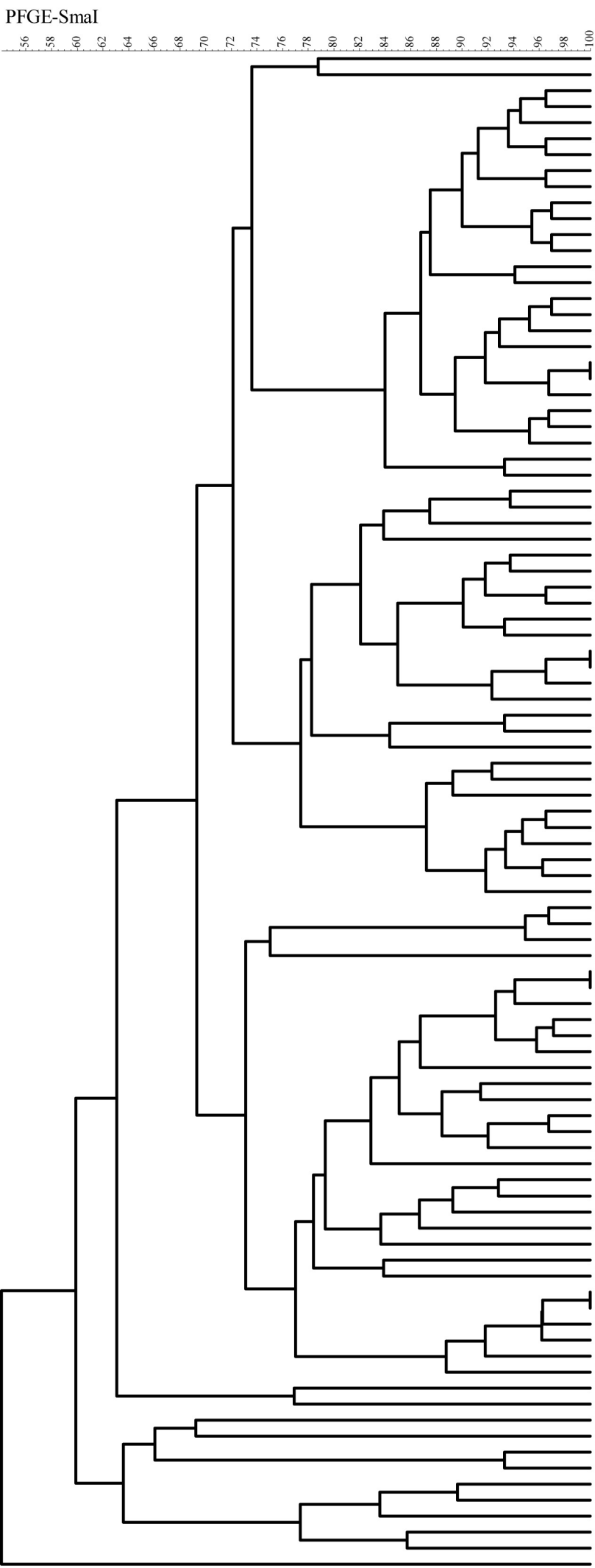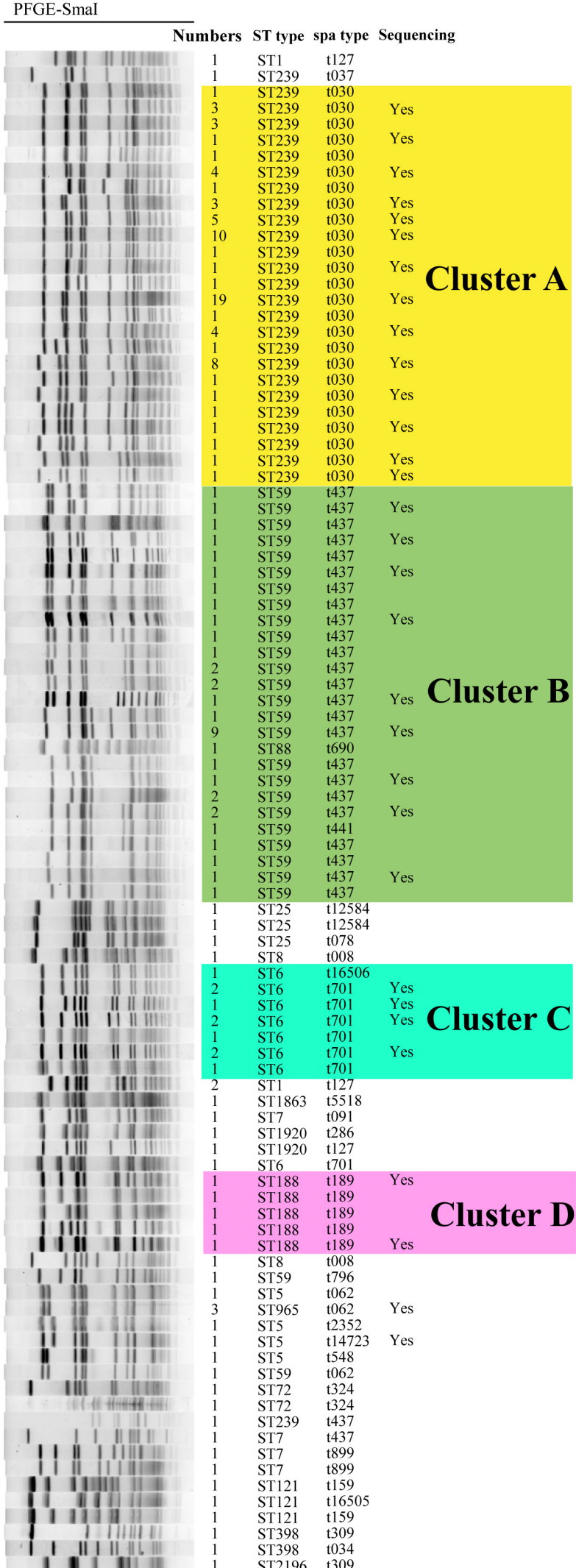

Supplement: Supplementary file 1 — Additional file 1. The molecular typing database of patients’ MRSA in Yunnnan province of China. [file 12879_2020_4866_MOESM1_ESM.pdf]

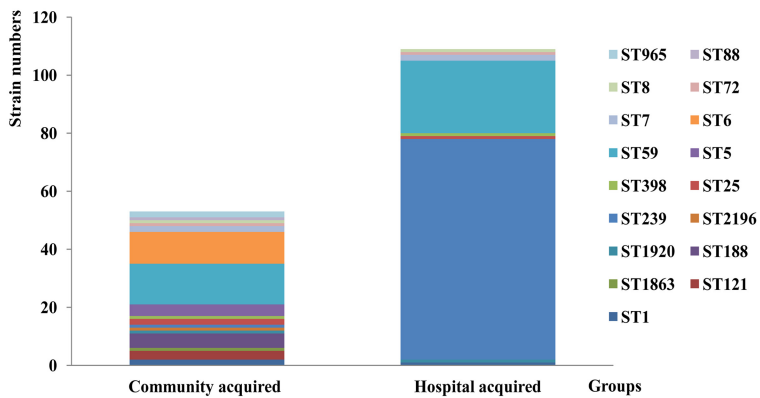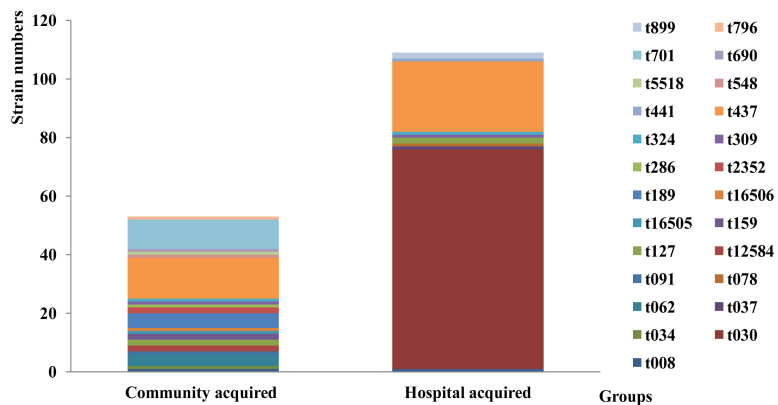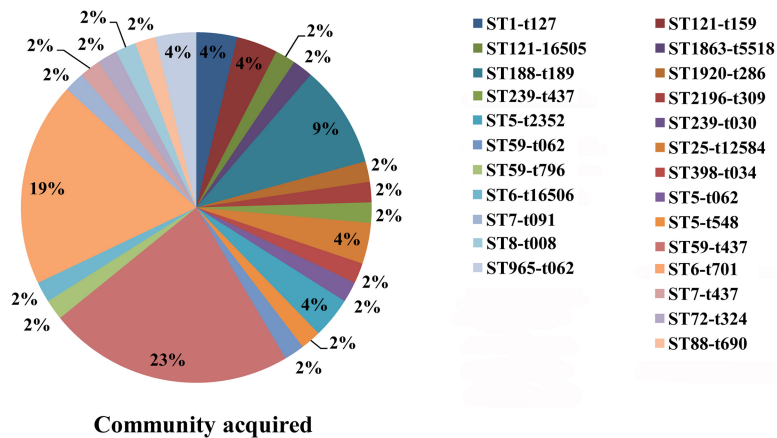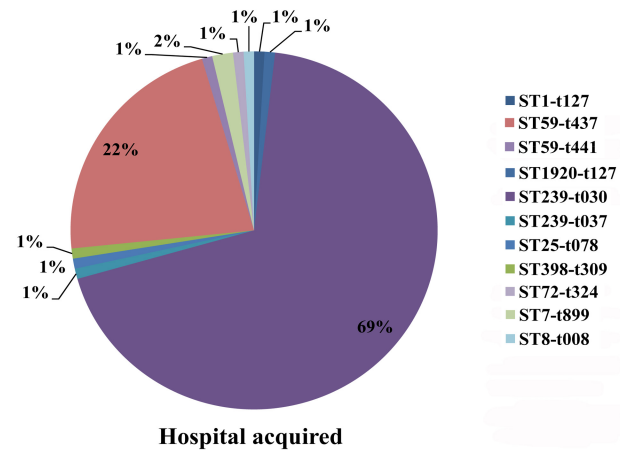

Supplement: Supplementary file 2 — Additional file 2. The genotype profiles of patients’ MRSA in Yunnan province of China. [file 12879_2020_4866_MOESM2_ESM.pdf]
